# Optimization path and implementation effectiveness of infection prevention and control interventions in a children’s hospital: a quantitative assessment

**DOI:** 10.3389/fpubh.2026.1721881

**Published:** 2026-02-13

**Authors:** Jing Hu, Yuwen Bao, Xiaorong Xiang, Shengfan Xue, Xu Wang, Qian Li

**Affiliations:** 1 Healthcare-Associated Infection Control Department, Children’s Hospital of Nanjing Medical University, Nanjing, Jiangsu, China; 2 Scientific and Technical Department, Children’s Hospital of Nanjing Medical University, Nanjing, Jiangsu, China; 3 President’s Office, Children’s Hospital of Nanjing Medical University, Nanjing, Jiangsu, China

**Keywords:** cleaning and disinfection, healthcare associated infection, isolation, quality control indicators, quantitative assessment, standard precaution, sterile operations

## Abstract

**Objective:**

This study aims to perform innovative quantitative assessments in managing healthcare-associated infections (HAIs) to pave the way for effective methods and pathways toward precise infection prevention and control (IPC).

**Methods:**

A total of 61 diagnosis and treatment departments of a tertiary grade A children’s hospital were included in this study. By establishing a quantitative assessment form, we determined the database of IPC assessment indicators and implemented basic IPC quantitative assessment practices. The pre-practice stage (January 2017-December 2020) was set as the control group, and the practice stage (January 2021-December 2024) was set as the experimental group. A comparison of quality control indicators related to HAIs was conducted between the two groups, along with the observation of trends in IPC compliance rates at the hospital level and in key departments following implementation.

**Results:**

The incidence rate of HAIs and the detection rate of multidrug-resistant organism infections in the experimental group were both lower than those in the control group, with *p*-values of <0.001 and <0.05, respectively. The hand hygiene compliance rate among healthcare staff significantly improved (*p* < 0.001). The implementation of IPC measures led to a yearly increase in compliance with standard precautions, cleaning, disinfection, sterilization, and isolation across the hospital, and these improvements were statistically significant (*p* < 0.001). The compliance rate of IPC interventions in high-risk departments increased annually (*p* < 0.01).

**Conclusion:**

The implementation of a quantitative assessment based on the quantified management of IPC interventions enabled the hospital and its departments to enhance the quality of their essential IPC management. It also improved the behavior of healthcare staff in implementing IPC and reduced the occurrence of HAIs.

## 1 Introduction

Healthcare-associated infections (HAIs) are a public health issue of global concern. An epidemiological study conducted by the World Health Organization (WHO) in 14 countries reported that the overall incidence of HAIs was 7.6% in developed countries and 10.1% in developing countries (1). Strengthening the implementation of healthcare-associated infection prevention and control (IPC) measures is an urgent necessity in hospitals. It is also essential to enhance the quality of medical care and public well-being (2, 3). Traditional IPC management approaches tend to be less standardized and more subjective, relying heavily on random inspections for evaluation. The assessment content is typically experience-based and lacks an objective, systematic, and reproducible evaluation framework. Thus, the efficiency and effectiveness of implementing IPC measures frequently fall short of expectations. Additionally, many medical institutions in China face numerous challenges in implementing IPC interventions (4). For instance, HAI management involves a wide range of responsibilities, complicated and diverse clinical workflows, and heterogeneous staff compositions (including physicians, nurses, and cleaning personnel) with varying levels of knowledge and skills in IPC (5, 6). It is critical to establish a systematic, quantifiable assessment framework for basic IPC interventions to improve the implementation of IPC measures and further enhance the level of refined management.

The *Guidelines on Core Components of Infection Prevention and Control Programs at the National and Acute Healthcare Facility Level* issued by the WHO serve as an essential guide for IPC practices. The guidelines clearly state the necessity for healthcare facilities to implement IPC programs and emphasize the importance of “water, sanitation, and hygiene” and “antimicrobial resistance reduction” (7). Since 2021, a tertiary Grade A children’s hospital has progressively explored the construction of a quantitative assessment system for IPC by integrating the WHO guidelines (8–10) with relevant national standards (11–13) pertaining to HAIs. IPC interventions were categorized into four dimensions: standard precautions, cleaning and disinfection management, isolation, and sterile operations. By comprehensively reviewing IPC interventions and crucial implementation components and organizing brainstorming sessions with infection prevention and control professionals and clinical department staff, we continuously addressed challenges related to indicator design and complex data collection. Subsequently, we developed an evaluation indicator scale. The final assessment results were determined by calculating the “compliance rate” based on the evaluation scale, thereby completing the quantitative evaluation.

Quantitative indicators for IPC assessments not only meet the daily needs of clinical departments for managing HAI prevention and control but also contribute to the optimization of management processes. On the one hand, they are characterized by assessability, precision, and incentivization. This system transforms abstract IPC behaviors into concrete numerical values, providing comparable and traceable data to support HAI management. Crucially, this data collection requires no specialized monitoring devices or complex information systems; instead, it enables HAI control departments to establish routine monitoring datasets. Furthermore, by implementing personalized quantitative IPC evaluations across different departments and facilitating timely feedback and corrective actions at both hospital and departmental levels, the system fosters a shift from an experience-based approach to quantitative management. On the other hand, implementing a quantitative assessment system provides a motivational driver for healthcare workers to adhere to and improve IPC intervention measures. By linking quantitative assessment results to performance evaluations, it stimulates proactive engagement and enthusiasm among staff in implementing IPC interventions.

In China, the implementation of IPC is still being explored, particularly in regional healthcare institutions and primary care facilities. This study established a quantitative assessment system for IPC interventions, which enhanced the execution capacity of clinical departments to implement IPC measures and provided a methodological pathway to advance precise IPC practices.

## 2 Materials and methods

### 2.1 Data sources

A tertiary Grade A children’s hospital has implemented innovative practices involving quantitative assessment of IPC in HAI management since 2021. The study focused on 61 diagnosis and treatment departments as the research objects, 16 of which were pivotal departments with a high risk of infection, such as the Neonatal Ward, the Intensive Care Unit (PICU, SICU, CCU), the Infectious Diseases Department, the Operating Suites, the Stomatology Department, and the Endoscopy Center (14); the remaining 45 departments were general infection control departments, such as General Surgery, Oncology, Neurosurgery, Gastroenterology, Cardiovascular Medicine, and Respiratory Medicine. We selected the pre-practice stage (January 2017–December 2020) as the control group, and the practice stage (January 2021–December 2024) was selected as the experimental group. All IPC data from both groups were obtained by infection prevention and control professionals (ICPs) through *Nosocomial Infection Surveillance* and real-time, on-site IPC surveillance.

A two-person verification process was conducted by ICPs for all collected data, ensuring that each entry was cross-checked. In cases of disagreement, senior experts from the Healthcare-Associated Infection Control Department conferred to reach a consensus, thereby guaranteeing both consistency in data evaluation and uniformity in data quality. The ICPs were internal staff members of the Healthcare-Associated Infection Control Department. Throughout the 8-year study period, the ICPs uniformly received training in IPC professional knowledge and skills from the provincial HAI management department and obtained the corresponding qualification certificates before conducting independent assessments. The hospital regularly organized training sessions for ICPs to conduct HAI training for clinical department staff, focusing primarily on high-risk areas and vulnerabilities in departmental infection control.

### 2.2 Methods

#### 2.2.1 Establishing a quantitative assessment form and assessment indicators

This study adhered to the relevant guidelines issued by the WHO and the National Health Commission of the People’s Republic of China (7–13). It also followed the principles of evidence-based medicine (15) and the characteristics of diagnosis and treatment in specialized children’s hospitals (16, 17). Through a literature review and policy analysis (18–21), IPC measures and implementation elements were selected and refined, covering four components: standard precautions, cleaning and disinfection management, isolation, and sterile operations. We organized IPC experts (e.g., physicians, nurses with master’s degrees in public health, or other relevant qualifications) who worked together with functional departments, such as the Medical Affairs Department, Nursing Department, and General Affairs Department, to establish an IPC quantitative assessment form after multiple rounds of revisions and refinements. The form summarized key elements for the four IPC components (Table 1). On this basis, the key points for the implementation of IPC interventions were further refined, and a total of 85 critical assessment indicators were initiated, including “standard precautions (20 items),” “cleaning, disinfection, and sterilization (25 items),” “isolation (20 items),” and “sterile operations (20 items),” forming four IPC assessment indicator databases, as presented in Supplementary Tables 1–4. Both key and general departments can effectively adapt to this IPC intervention assessment system. Moreover, the 85 evaluation items comprehensively addressed potential HAI risks across the continuum of clinical care, thereby supporting daily departmental IPC requirements.

**Table 1:** IPC quantitative assessment form.

Assessment indicators	Contents and main points of assessment
Standard precautions	Provision of personal protective equipment (PPE). Compliance with personal protective measures. Standardization of the implementation of hand hygiene. Standardization of the implementation of safe injections. Proper and timely management of occupational exposures.
Cleaning, disinfection, and sterilization	Strict adherence to principles of cleaning, disinfection, and sterilization Proper use of disinfectants and cleaning products Implementation of cleaning and disinfection requirements according to the risk level of the clinical environment and medical items. Standardization and implementation of management requirements, such as the preparation, monitoring, and registration of disinfectants. 5. Standardization of the use and management of cleaning products.
Isolation	Selection and implementation of appropriate isolation measures based on the type of patient and the risk of disease transmission: Timely isolation of issues. Correct choice of isolation methods. Standardization of isolation identification. Effective implementation of education for patients and accompanying personnel.
Sterile operations	Ensuring that the environment, items, and healthcare staff are prepared to meet the specification requirements before any procedure. Strict adherence to sterile operation principles during procedures. Code development for the disposal of operating parts, medical waste, and reusable items after procedures.

#### 2.2.2 Implementing basic IPC quantitative assessment practices

Currently, our hospital maintains a staffing ratio of one full-time infection prevention and control professional (ICP) per 200 inpatients. Each ICP is responsible for a defined set of departments, ensuring clear and non-overlapping jurisdictional boundaries. Based on the key elements of the quantitative IPC assessment (Table 1) and the corresponding 85 IPC intervention evaluation indicators (Supplementary Tables 1–4), we conducted quantitative assessments at both the hospital and department levels. The assessment frequency was as follows: Departments with a high infection risk adopted monthly coverage, and general departments adopted quarterly full coverage.

The hospital-level assessment was mainly conducted to monitor and evaluate the implementation of IPC interventions in clinical departments by ICPs. A binary scoring system was used to evaluate IPC assessment indicators across the four groups. If the execution of an indicator met the standard, the pass count was increased by one. Therefore, the compliance rate was defined by the following formula: (Number of compliant intervention indicators assessed)/(Total number of intervention indicators assessed). The implementation indicators for IPC interventions across the four groups were selected from their respective indicator databases. Based on the execution status (compliant/non-compliant), the compliance rate was calculated separately for each group. The calculation formula and passing criteria are as follows.

1. Compliance rate of standard precaution implementation = (Number of compliant standard precaution intervention indicators assessed)/(Total number of standard precaution intervention indicators assessed) × 100% (Compliance rate ≥95%).
2. Compliance rate of cleaning, disinfection, and sterilization implementation = (Number of compliant cleaning, disinfection, and sterilization intervention indicators assessed)/(Total number of cleaning, disinfection, and sterilization intervention indicators assessed) × 100% (Compliance rate ≥ 95%).
3. Compliance rate of isolation implementation = (Number of compliant isolation intervention indicators assessed)/(Total number of isolation intervention indicators assessed) × 100% (Compliance rate 100%).
4. Compliance rate of sterile operation implementation = (Number of compliant sterile operation intervention indicators assessed)/(Total number of sterile operation intervention indicators assessed) × 100% (Compliance rate 100%).

Any department with a compliance rate below the established standard received a formal rectification request. Subsequent supervision included enhanced monitoring of deficient dimensions, as exemplified by increased audit frequency or a broader scope of indicators. Different healthcare institutions should dynamically adjust the indicators of the four groups based on their foundational levels and modify the corresponding compliance rate standards.

The departmental-level quantitative assessment primarily focused on internal self-monitoring by healthcare workers within the department. Rectification efforts were aimed at addressing the issues identified through feedback from ICPs during the basic and dynamic assessments. Departments were required to strengthen the implementation of inadequately applied IPC measures in their daily work. Dynamic assessments targeted departments with substandard compliance rates in IPC intervention implementation during the basic assessments. Based on their clinical characteristics and identified IPC weaknesses, tailored indicators were selected from the IPC intervention assessment indicator datasets to generate personalized assessment forms for use in the next stage. For example, during the initial phase of the quantitative assessment implementation, the compliance rates for IPC measures, such as standard precautions in the neonatal ward, cleaning and disinfection in the CCU, and isolation in the PICU, were relatively low. By implementing personalized crucial assessments and dynamically adjusting evaluation indicators based on practical conditions at each stage, improvements in these areas were effectively promoted.

#### 2.2.3 Application of assessment results

The quantitative assessment results of the “IPC intervention compliance rate” for each department, along with other relevant HAI surveillance data, would be fed back to the clinical departments monthly or quarterly to drive continuous improvement. These data were also reported to the Medical Quality Management Department and linked to the departmental performance assessment. In addition, based on the annual performance review, which included comprehensive HAI surveillance data and basic IPC quantitative assessments, outstanding departments and individuals were identified. They received the “Excellent Department Award for Infection Control” and “the Guardian Award for Infection Control,” respectively.

### 2.3 Evaluation criteria

The control and experimental groups were compared based on the quality control indicators related to healthcare-associated infections (22), including the incidence rate of HAIs, the detection rate of multidrug-resistant organism infections, the hand hygiene compliance rate of hospital staff, and the infection rate of clean wound surgical sites. The specific calculation formulas are as follows.

① Incidence rate of HAIs = (Number of cases of new HAIs)/(Total number of hospitalized patients during the same period) × 100%.

② Detection rate of multidrug-resistant organism infection = (Number of cases of multidrug-resistant organism infection)/(Total number of hospitalized patients during the same period) × 100% [Multidrug-resistant organisms mainly include Carbapenem-Resistant Enterobacterales (CRE), Carbapenem-Resistant Klebsiella (CRKP), Carbapenem-Resistant *Acinetobacter Baumannii* (CRAB), Carbapenem-Resistant *Pseudomonas Aeruginosa* (CRPA), Methicillin-Resistant *Staphylococcus Aureus* (MRSA), and Vancomycin-Resistant Enterococci (VRE).]

③ Compliance rate of hospital staff hand hygiene = (Number of hand hygiene opportunities actually performed by the surveyed hospital staff)/(Total number of hand hygiene opportunities during the same survey period) × 100%.

④ Infection rate of a clean wound surgical site = (Number of infection cases following clean incision surgery among inpatients)/(Total number of clean incision surgical operations among inpatients during the same period) × 100% (This indicator counts surgical site infections occurring within 30 days after implant-free clean wound surgery, or within 1 year if an implant is present.)

On-site supervision was conducted, followed by the evaluation and analysis of IPC practices. This allowed for tracking the annual trend in the compliance rate of basic IPC measures at the hospital and high-risk departmental levels.

### 2.4 Statistical analysis

Data analysis was performed using the Statistical Package for Social Sciences (SPSS, IBM, NY, United States). The counting data were statistically described using percentages and rates. The chi-square test was used to compare the two groups, and the chi-square trend test was used for multiple time-point comparisons. Statistical significance was set at *p* < 0.05.

## 3 Results

### 3.1 Comparison of the changes in the related quality control indicators of HAIs between the control group and the experimental group

The incidence of HAIs in the experimental group was significantly lower than that in the control group (*p* < 0.001). The detection rate of multidrug-resistant organism infection was also lower in the experimental group than in the control group (*p* < 0.05). Hospital staff compliance with hand hygiene increased sharply (*p* < 0.001). In addition, no statistically significant decrease in the infection rate at clean wound surgical sites was observed in the experimental group compared with that in the control group. This is likely because the baseline infection rate in the hospital consistently remained very low (Table 2). In other words, the rate remained substantially below the 1.5% threshold set by the *Hospital Management Evaluation Guidelines* (23).

**Table 2:** Comparison of the changes in the related quality control indicators of HAIs between the control and experimental groups.

Related quality control indicators of HAIs (%)	Control group					Experimental group					*χ* ^2^	*p*
	2017	2018	2019	2020	Total	2021	2022	2023	2024	Total		
Incidence rate of healthcare-associated infection	2.29	2.12	1.95	1.82	2.05	1.69	1.45	1.36	0.95	1.37	481.97	<0.001
Detection rate of multidrug-resistant organism infection	0.047	0.045	0.069	0.046	0.052	0.037	0.056	0.042	0.034	0.042	4.080	<0.05
Hospital staff compliance with hand hygiene	58.45	61.90	69.43	81.65	72.94	86.22	87.91	88.50	85.86	87.10	819.80	<0.001
Infection rate of clean surgical wound sites	0.13	0.20	0.13	0.07	0.13	0.16	0.12	0.15	0.12	0.14	0.19	0.667

### 3.2 Comparative trends in IPC compliance between whole-hospital and high-risk departments after quantitative assessment implementation

Since implementation, the compliance rate of IPC measures at the hospital has shown an increasing trend each year. The compliance rates for standard precautions, cleaning, disinfection, sterilization, and isolation implementation have increased annually, and the difference was statistically significant (*p* < 0.001). Although the improvement in sterile operation compliance rates s was not statistically significant, they remained consistently within a stable range of 98.78 to 99.39% over the 4-year period, as shown in Table 3. Some high-risk departments, such as the neonatal ward, CCU, and PICU, have dramatically improved their IPC weaknesses. The compliance rate of relevant IPC indicators has shown an increasing trend over the years. This trend was statistically significant (*p < 0.01*), as shown in Table 4.

**Table 3:** Changing trends of IPC compliance rates in the whole hospital.

Assessment indicators (%)	2021	2022	2023	2024	*χ* ^2^	*p*
Compliance rate of standard precaution implementation	95.77	96.85	98.41	98.65	124.40	<0.001
Compliance rate of cleaning, disinfection, and sterilization implementation	85.81	94.48	96.24	97.60	590.85	<0.001
Compliance rate of isolation implementation	88.63	95.55	96.76	98.00	196.19	<0.001
Compliance rate of sterile operation implementation	99.23	99.18	99.39	98.78	0.728	0.394

**Table 4:** Changing trends of IPC compliance rates in high-risk departments.

Assessment indicators (%)	2021	2022	2023	2024	*χ* ^2^	*p*
Compliance rate of standard precautions in the neonatal ward	94.70	97.50	99.38	99.59	13.438	<0.001
Compliance rate of cleaning, disinfection, and sterilization in the CCU	87.50	97.30	92.90	97.97	8.1 00	<0.01
Compliance rate of isolation in the PICU	93.15	96.98	97.95	98.43	9.561	<0.01

## 4 Discussion

The core of healthcare-associated infection management lies in preventing patients and healthcare staff from being harmed by avoidable infections through the implementation of IPC measures. IPC also plays a crucial role in safeguarding medical safety and quality (15, 24). In the past, medical institutions paid insufficient attention to IPC, and the professional competence of assessors in the IPC interventions has varied significantly. Despite guidance from IPC guidelines and HAI standards, evaluation of IPC interventions was often based on the experiential judgment of assessors. This led to unclear assessment priorities, cumbersome inspection items, and low IPC efficiency (1). In recent years, however, Children’s Hospital of Nanjing Medical University has pioneered a quantitative IPC assessment system covering standard precautions, cleaning and disinfection, isolation, and sterile operations. This initiative was driven by a heightened emphasis on IPC from policies and institutions, and the catalytic effect of the COVID-19 pandemic (25). This assessment system was multidimensional and multilevel. It was aligned with authoritative domestic and international guidelines, including those from the WHO and the National Health Commission of China (7–13). It was also informed by real-world IPC experience in the hospital setting. Through this process, four groups and 85 crucial implementation checkpoints were identified, ranging from the macro-level quantitative metric of “compliance rate” to the micro-level specific evaluation criteria. This structurally complete, logically rigorous, and highly systematic assessment framework enables comprehensive and precise monitoring of IPC implementation in the hospital examined, thereby contributing to measurable improvements in healthcare workers’ compliance behaviors with IPC measures.

Specifically, after implementing a quantitative assessment system for measuring IPC intervention compliance, the incidence of HAIs in our hospital decreased from 1.69% in 2021 to 0.95% in 2024. A cross-sectional study conducted across 5,736 healthcare institutions in China during the same period reported that the national incidence of HAIs in 2024 ranged from 0.66 to 2.35% (26). HAI incidence at our hospital was among the lowest in the country. Furthermore, hand hygiene compliance among our medical staff improved by 14.16% over the 4-year period. In comparison, a medical institution in Finland achieved a significant enhancement in hand hygiene compliance among its doctors and nurses, with an increase of 12.1% over 5 years starting in 2013, simply by introducing direct observation of hand hygiene practices (27). Our findings are further supported by research from Ershova et al. (28), in which they implemented an IPC program in a neuro-ICU combining education, IPC measures, and monitoring integrated with antimicrobial stewardship. Their study indicated that personalized IPC interventions significantly contribute to HAI prevention and reduce antimicrobial resistance. Similarly, the implementation of targeted IPC interventions in our study, which focused on standard precautions in the Neonatal Ward, cleaning and disinfection in the CCU, and isolation practices in the PICU, resulted in significant improvements. Compliance rates in these areas increased by 4.89, 10.47, and 5.28%, respectively.

This study also demonstrated the significance of HAI management, which can be observed at four levels. First, by implementing differentiated assessment strategies and formulating different assessment plans for high-risk departments and general departments. This measure not only highlighted IPC work in crucial departments but also took the integrity of the entire hospital’s infection control management into account, realizing accurate resource delivery. Second, personalized assessments were conducted based on problem orientation, and assessment indicators were dynamically adjusted according to the weaknesses and implementation loopholes of each department. Simultaneously, this approach aimed to solve outstanding problems and formed a closed loop of efficient rectification through precise intervention. Thus, we improved the targeting and effectiveness of the infection control. Third, a closed-loop management mechanism of “assessment-feedback-improvement” was constructed, and the assessment results were fed back to the head of the various clinical departments in a timely manner, which was deeply linked with the performance of the departments. It not only helped departments formulate scientific, continuous improvement plans by analyzing high-infection-risk links (29) but also significantly enhanced basic infection control management by fully mobilizing staff enthusiasm through a transparent incentive mechanism (30). Moreover, by establishing a standardized assessment system, this study clarified the roles of relevant departments and personnel and aligned their understanding of fundamental IPC standards, thus providing clear objectives and actionable guidance for HAI prevention efforts.

The COVID-19 pandemic has posed new challenges and increased the need for hospital infection prevention and control (31). The rapid deployment of this quantitative assessment mechanism was aided by the increased attention to infection control within medical institutions during the COVID-19 pandemic. After 4 years of implementation at both the hospital and department levels, key infection indicators showed sustained improvement, with significant increases in IPC compliance rates both hospital-wide and in high-risk departments. Since the normalization of the management of epidemic prevention and control of COVID-19 in 2023, all indicators have continued to improve, which verified the effectiveness and stability of the quantitative assessment practices and explored a set of more efficient implementation paths for the accurate prevention and control of healthcare-associated infections.

However, this study has certain limitations. First, this study was based on a single children’s hospital. Although the core requirements for HAI prevention and control are clearly defined, the indicators for IPC interventions need to be dynamically adapted to the characteristics of different hospitals. Second, the current practices lacked AI-based informatization tools. Third, although the quantitative IPC assessment system did not require specialized equipment and had low overall implementation costs, this study was limited to intra-hospital practices and relied on relatively homogeneous data, which limited its ability to robustly demonstrate applicability in low-resource settings. Finally, our IPC practices were implemented during the COVID-19 period. Although our hospital was not a designated pandemic response facility, the potential influence of the pandemic cannot be entirely ruled out. In the future, we will continue to implement a quantitative IPC assessment system while expanding our collaboration with other health institutions. Our future practices will extend to peer-level medical facilities, primary care settings, and low-resource regions to gather more diverse evidence for validation. Furthermore, efforts will be made to integrate a quantitative assessment system with artificial intelligence to continuously optimize the hospital IPC framework, thereby enhancing its potential for broader dissemination.

## 5 Conclusion

Our study established and implemented a quantitative assessment system, allowing for both routine oversight in general departments and targeted precision management in key departments, which promoted improved compliance with IPC interventions. This has also led to a reduction in the incidence of HAIs and an improvement in IPC behavior among staff. Over 4 years of practice, consistent improvement across indicators has demonstrated the effectiveness and stability of this quantitative assessment system. In addition, it explored a relatively efficient implementation pathway for precise HAI prevention and control, providing valuable experience for other healthcare institutions to promote quantifiable IPC management.

## Data Availability

The raw data supporting the conclusions of this article will be made available by the authors, without undue reservation.
