# Supplementary material for: Optimization path and implementation effectiveness of infection prevention and control interventions in a children’s hospital: a quantitative assessment
Source: Front Public Health. 2026 Feb 13;14:1721881. doi: 10.3389/fpubh.2026.1721881 (PMC12946133; doi:10.3389/fpubh.2026.1721881)
Supplement: Supplementary file 1 [file Supplementary_file_1.docx]

**Additional file：**

**Supplementary Table 1** Assessment indicators of standard precaution interventions

| **Contents of assessment** | **Assessment indicators** |
| --- | --- |
| 1. Provision of personal protective equipment (PPE) | 1.1 PPE should be placed at fixed, designated points within the clinical department and maintained in complete stock. |
|  | 1.2 PPE should be free from expiration, physical compromise, or contamination. |
| 1. Compliance with personal protective measures | 2.1 A medical mask should be used when making routine clinical interactions (e.g., ward rounds, vital sign monitoring) with general patients without infectious diseases. |
|  | 2.2 A medical mask (N95 and higher-level respiratory protection equipment should be worn for airborne transmission), a disposable medical cap, disposable latex gloves, and an isolation gown should be used when contacting with infectious, protective isolation, or MDRO-positive patients. |
|  | 2.3 A medical mask, a disposable medical cap, and gloves (select the appropriate gloves based on the specific situation) should be used when performing sterile operations. |
|  | 2.4 A medical mask, a disposable medical cap, sterile surgical gloves, an isolation gown, and a face shield or goggles should be used when performing fiberoptic bronchoscopic and gastrointestinal endoscopic examinations or treatments. |
|  | 2.5 A medical mask, a disposable medical cap, sterile surgical gloves, scrubs and a sterile surgical gown should be used when performing surgical operations. |
|  | 2.6 A medical mask, a disposable medical cap, disposable latex gloves, and an isolation gown (a face shield or goggles if necessary) should be used when performing high-splash-risk procedures, such as enema administration, throat swab sampling, and oral manipulations. |
|  | 2.7 A medical mask, a disposable medical cap, sterile surgical gloves and a sterile isolation gown when performing PICC, deep vein catheterization and renal puncture procedure. |
| 1. Hand hygiene | 3.1 Hand hygiene facilities should be adequate and available. |
|  | 3.2 Alcohol-based hand rub must be labeled with its expiration date in use. |
|  | 3.3 Hand hygiene timing is appropriate. |
|  | 3.4 Hand hygiene method is correct. |
| 1. Safe injection practices | 4.1 Disposable sterile syringes and their needles should be “one person, one needle, one tube and one use”. |
|  | 4.2 Withdrawn medications and opened sterile intravenous fluids must not be used if left standing for more than 2 hours. |
|  | 4.3 Any solvent that has been unsealed and withdrawn into a syringe should be discarded after 24 hours. |
|  | 4.4 Recap a needle using both hands and manual disassembly of sharp items are forbidden. Touch used needles, blades, or any sharp items directly with bare hands is also forbidden. |
|  | 4.5 Contaminated sharps should be discarded directly into designated sharps containers. The container must be closed and sealed as soon as it is filled to the ¾ capacity mark. |
| 1. Occupational exposure | 5.1 Extra precautions must be applied according to the transmission route of the pathogen. |
|  | 5.2 Healthcare staff shall be trained and competent in knowledge following occupational exposure. |

**Supplementary Table 2** Assessment indicators of cleaning, disinfection and sterilization interventions

| **Contents of assessment** | **Assessment indicators** |
| --- | --- |
| 1. Principles of cleaning, disinfection and sterilization | 1.1 The principle of cleaning before disinfection, as well as wet sanitation should be adopted. |
|  | 1.2 The cleaning of wards and consulting rooms should follow these guiding principles: zoned progression, sequential order (top-to-bottom, inside-to-outside, mild pollution-to-severe pollution), and a unitized methodology. |
|  | 1.3 Personal protection should be taken when implementing cleaning and disinfection, and hand hygiene and personnel hygiene should be done at the end of work. |
|  | 1.4 Barrier containment measures can be taken for high-touch surfaces that are easily contaminated and difficult to clean and disinfect. The barrier covers (e.g., plastic films) used for this purpose should be replaced after each use. |
|  | 1.5 Spot cleaning and disinfection should be performed immediately when surfaces contaminated with patient body fluids, blood, or similar substances during diagnosis or treatment. |
|  | 1.6 Reusable items must be “disinfected or sterilized after each person use”. |
|  | 1.7 Disposable medical devices should not reused. |
| 1. Disinfectants and cleaning products | 2.1 All types of disinfectants should be used within the expiry date specified in the product instructions, with both the opening date and expiration date clearly indicated |
|  | 2.2 Select appropriate disinfectants according to the characteristics of the pollution pathogens. Ensure that the preparation, concentration, monitoring, and usage methods of the disinfectants are correct. |
|  | 2.3 Containers used for storing disinfectants should meet the required standards of disinfection or sterilization. |
|  | 2.4 All types of cleaning and disinfection products should be adequately equipped and used in designated zones. |
|  | 2.5 Used or contaminated wiping towels should not be re-immersed into clean water, detersive, or disinfectants after use. |
|  | 2.6 The dirty washing room and disinfection room should be kept clean and tidy without any stored clutter. |
| 1. Medical environment | 3.1 Different frequency of environmental cleaning and disinfection should be implemented according to different risk levels: 1-2 times/day in low-risk areas, 2 times/day in medium-risk areas, and ≥2 times/day in high-risk areas. |
|  | 3.2 The ward should ventilated at least twice a day for no less than 30 minutes each time. Ultraviolet-ray-lamp or air disinfecting machine should be uesd and recorded every day when the ventilation is poor. |
|  | 3.3 Ultraviolet-ray-lamp, air disinfecting machine, outlet/return port of air conditioner and filters should be regularly cleaned and maintained, with records of such cleaning and maintenance established. |
|  | 3.4 The surface properties should be considered when cleaning or disinfecting. The smooth surface should be wiped with a suitable disinfectant or irradiated with ultraviolet rays at close range; The surface of porous materials should be disinfected by soaking or spraying; Disinfection wet wipes can be used for cleaning and disinfection when there is no obvious pollution. |
|  | 3.5 Intensive cleaning and disinfection should be implemented when there is an outbreak of healthcare associated infection (such as *Acinetobacter*, *Clostridium difficile* and *Norovirus)* or multi-drug resistant bacteria are detected on environmental surfaces . |
| 1. Medical items | 4.1 Critical items should be sterilized before use; semi-critical items should achieve high-level disinfection or intermediate-level disinfection before use; no-critical items should select intermediate-level/low-level disinfection or keep cleaning before use. |
|  | 4.2 Appropriate disinfectants should be selected in accordance with the product instructions for the medical instruments and equipment used in various specialties (e.g., flexible endoscopes, ventilators and their accessories, monitors). |
|  | 4.3 Sterile items should be removed from the outer packaging box and stored in the sterile warehouse shelves. They should be placed in the order of sterilization dates and marked. They also should not be mixed with non-sterile items. The placement should meet the requirements of 50-20-5 (cm). |
|  | 4.4 Clean medical textiles should be stored in clean containers or cabinets in the cleaning fabric warehouse in the ward, with obvious marking. |
|  | 4.5 Medical textiles should be classified and collected after use (e.g., soiled and foul textile, infected textiles, and neonatal textiles). |
| 1. Monitoring | 5.1 Regularly environmental hygiene sampling and monitoring should be performed in the clinical department. If it is unqualified, it should timely feedback to Healthcare Associated Infection Control Department or infection prevention and control (IPC) professionals. Then, professionals will guide the implementation of IPC interventions. |
|  | 5.2 Regular monitoring of cleaning and disinfection should be performed using methods such as visual inspection, ATP detection, fluorescent marking, and microbial culture. |

**Supplementary Table 3** Assessment indicators of isolation interventions

| **Contents of assessment** | **Assessment indicators** |
| --- | --- |
| 1. Timely and correct isolation | 1.1 Isolation orders must be prescribed correctly and promptly within 36 hours of receiving pathogen-specific results or confirming a diagnosis, guided by the identified transmission routes. |
|  | 1.2 Correct isolation methods should be applied:  ①For confirmed infectious diseases and multi-drug resistant organism (MDRO): Single‑room isolation is preferred. Patients with airborne transmission diseases should be placed in negative‑pressure rooms. When resources are limited, cohort isolation of patients with the same pathogen is acceptable, with a bed spacing of ≥1.2m. Patients with MDRO infection or colonization should not be roomed with patients who have endotracheal intubation, deep venous catheterization, open wounds, or compromised immunity.  ②For suspected infectious diseases: single‑room isolation is required. |
|  | 1.3 Individuals with close exposure to an infectious source should be placed under medical observation based on the characteristics of the pathogen and must not be transferred to a non-isolation observation room. |
|  | 1.4 Isolation signage must be clearly visible and correctly implemented. |
|  | 1.5 Personnel access should be restricted. |
| 1. Management of healthcare personnel in clinical units | 2.1 Department staff (e.g., physician, nurse, and cleaning personnel) should be aware of the situation of isolated patients in their units (such as bed number and type of isolation required). |
|  | 2.2 When conditions permit for rounds, diagnosis, nursing procedures, and cleaning or disinfection, the order should be followed: general patients first, followed by isolated patients (with protective isolation patients preferably prioritized). |
|  | 2.3 Suitable and essential PPE must be available in accordance with relevant national regulations. It should also be maintained within expiry dates, and stored for easy retrieval. |
|  | 2.4 Department staff in clinical units must select PPE according to the transmission route of the pathogen and adhere to standardized utilization procedures.  standardized usage protocols. |
|  | 2.5 Hand hygiene must be strictly performed with correct timing and methods. Hand-washing with running water should be selected when contacting microorganisms that are less susceptible to alcohol‑based hand rub (such as *Clostridioides difficile* and *norovirus* ). |
| 1. Management of items and the environment | 3.1 Medical device and items should be specially used or disinfected after each use. |
|  | 3.2 Bedside consumables should be placed appropriately according to the needs of diagnosis and treatment. |
|  | 3.3 Frequency of cleaning and disinfection among air and environmental surfaces, as well as effective ventilation should be increased: at least three times a day. Concentration of disinfectant should also be increased if necessary. Patients with intestinal infectious diseases should use the toilet to flush, and items contaminated by excrement should be disposed of in time. |
|  | 3.4 Terminal cleaning and disinfection should be performed after patient discharge. Reusable items (e.g., medical instruments, cubicle curtains) must undergo rigorous cleaning and disinfection. |
| 1. Management of medical wastes and medical textiles | 4.1 Domestic wastes and medical wastes produced by patients should be gathered in double‑walled yellow infectious waste bags, closed using the goose‑neck method with layered fastening to guarantee integrity and containment, and moved under closed conditions. |
|  | 4.2 Medical textiles should be gathered using designated orange bags for infectious linen and conveyed in a sealed manner. |
| 1. Management of patients and accompanying persons | 5.1 Visitation should be restricted, patients’ movement range should be limited, and health education should be provided to accompanying caregivers (including hand hygiene, PPE use, and waste handling). |
|  | 5.2 Auxiliary examinations should be performed at the bedside whenever possible, and examiners must take appropriate protective measures during bedside procedures. |
|  | 5.3 The receiving department/unit should be notified to implement effective measures when a patient requires external examinations, treatments, surgery, inter-departmental transfers or transportation. Healthcare staff and the patient must conduct appropriate protection, and timely disinfection should be performed. Medical masks should be provided to patients with respiratory infectious diseases and their accompanying persons. |
| 1. Monitoring and reporting | - 1. Attention should be paid to the occurrence of new similar cases within the unit. If there is a clustering of healthcare-associated infection (HAI) cases, suspected outbreaks, or trends indicating an outbreak, the situation must be promptly reported to the HAI control department. |

**Supplementary Table 4** Assessment indicators of sterile operations interventions

| **Contents of assessment** | **Assessment indicators** |
| --- | --- |
| 1. Before operation | 1.1 The treatment room is the primary site for sterile operations. When the patient's condition prohibits this, operations should be conducted at the bedside with the mandatory use of a treatment cart. |
|  | 1.2 The operational environment should be kept clean. The treatment room should be disinfected in accordance with established protocols, with records maintained, and all cleaning must be suspended 30 minutes before the operations. |
|  | 1.3 All items should be adequately prepared to avoid cross-contamination. |
|  | 1.4 All items should be properly sealed and remain within the expiration date. |
|  | 1.5 The treatment cart should be kept clean. Items should be arranged in a logical layout: the upper tier for sterile/clean materials, the lower tier for contaminated or post-use articles. |
|  | 1.6 Personal Protective Equipment (PPE) guidelines for clinical procedures: A medical mask, a disposable medical cap, and sterile surgical gloves should be uesd for operations including but not limited to bone marrow aspiration, thoracentesis, lumbar puncture, abdominal paracentesis, and wound dressing. Sterile isolation gowns must be added for central venous catheter insertion, renal biopsy, and liver biopsy. |
|  | 1.7 Healthcare staff should perform proper hand hygiene at the right moments. |
|  | 1.8 The patient should be placed in a suitable position to ensure proper exposure of the operation site, followed by cleansing of the area if indicated. |
| 1. Operation | 2.1 The disinfectants should be selected properly and within expiry date, and the blood culture draws should followed the three-step (alcohol–iodine tincture–alcohol) disinfection method. |
|  | 2.2 Surgical site disinfection should be performed in the correct direction. For clean incisions: from the center of the incision site outward to the periphery; For contaminated incisions (e.g., infected wounds or perineal area): from the periphery inward toward the incision or wound center. |
|  | 2.3 Disinfection methods should be performed correctly:  ①Injections and punctures: Two consecutive spiral disinfections should be performed from the center outward, covering a minimum area of 5cm × 5cm without skin gaps.  ②Central Venous Catheter Insertion: An area, which diameter >15cm or extend beyond the standard dressing dimensions (10cm × 12cm), should be disinfected.  ③Surgical/incision care: The surgical field should be disinfected with a ≥15 cm margin; The postoperative incision should be disinfected plus a 3-5cm peripheral zone (or exceeding the gauze/dressing coverage). |
|  | 2.4 Both the frequency of disinfectant applications and the contact (dwell) time should be appropriate and in accordance with established protocols. |
|  | 2.5 The maximal sterile barrier technique should be maintained for sterile operations (such as PICC and arterial/venous cannulation). For specified procedures, sterile fenestrated drapes must be used to avoid contamination risks. |
|  | 2.6 Sterile operations should be performed via an appropriate sequence and accurate method, maintaining strict adherence to sterile principles to avoid compromising sterile fields. |
|  | 2.7 Healthcare staff must adhere to proper hand hygiene timing and methodology, including performing hand hygiene immediately before putting on and after taking off sterile surgical gloves. |
| 1. After operation | 3.1 The patient and the target site for the operations should be properly prepared and handled. |
|  | 3.2 Medical wastes must be classified accurately at the point of generation, with no secondary sorting permitted. |
|  | 3.3 Reusable items should be placed in a sealed place for washing after initial decontamination. |
|  | 3.4 The treatment cart should be restocked and cleaned after use. |
|  | 3.5 Healthcare staff must adhere to proper hand hygiene timing and methodology. |
